# Rescue of Outer Hair Cells with Antisense Oligonucleotides in Usher Mice Is Dependent on Age of Treatment

**DOI:** 10.1007/s10162-017-0640-x

**Published:** 2017-10-12

**Authors:** Abhilash Ponnath, Frederic F. Depreux, Francine M. Jodelka, Frank Rigo, Hamilton E. Farris, Michelle L. Hastings, Jennifer J. Lentz

**Affiliations:** 1 Neuroscience Center of Excellence, Louisiana State University Health Sciences Center, 2020 Gravier Street, 8th Floor, New Orleans, LA 70112 USA; 2 Department of Cell Biology and Anatomy, Chicago Medical School, Rosalind Franklin University of Medicine and Science, 3333 Green Bay Rd, North Chicago, IL 60064 USA; 3 Ionis Pharmaceuticals, 2855 Gazelle Court, Carlsbad, CA 92010 USA; 4 Department of Cell Biology and Anatomy, Louisiana State University Health Sciences Center, 1901 Perdido Street, New Orleans, LA 70112 USA; 5 Department of Otolaryngology and Biocommunications, Louisiana State University Health Sciences Center, 533 Bolivar Street, New Orleans, LA 70112 USA

**Keywords:** Usher syndrome, hearing loss, *USH1C*, c.216G>A mutation, harmonin, antisense oligonucleotides, outer hair cells, DPOAE, inner hair cells, ABR, splicing, RNA

## Abstract

The absence of functional outer hair cells is a component of several forms of hereditary hearing impairment, including Usher syndrome, the most common cause of concurrent hearing and vision loss. Antisense oligonucleotide (ASO) treatment of mice with the human Usher mutation, *Ush1c* c.216G>A, corrects gene expression and significantly improves hearing, as measured by auditory-evoked brainstem responses (ABRs), as well as inner and outer hair cell (IHC and OHC) bundle morphology. However, it is not clear whether the improvement in hearing achieved by ASO treatment involves the functional rescue of outer hair cells. Here, we show that *Ush1c* c.216AA mice lack OHC function as evidenced by the absence of distortion product otoacoustic emissions (DPOAEs) in response to low-, mid-, and high-frequency tone pairs. This OHC deficit is rescued by treatment with an ASO that corrects expression of *Ush1c* c.216G>A. Interestingly, although rescue of inner hairs cells, as measured by ABR, is achieved by ASO treatment as late as 7 days after birth, rescue of outer hair cells, measured by DPOAE, requires treatment before post-natal day 5. These results suggest that ASO-mediated rescue of both IHC and OHC function is age dependent and that the treatment window is different for the different cell types. The timing of treatment for congenital hearing disorders is of critical importance for the development of drugs such ASO-29 for hearing rescue.

**Electronic supplementary material:**

The online version of this article (10.1007/s10162-017-0640-x) contains supplementary material, which is available to authorized users.

## **INTRODUCTION**

Usher syndrome (Usher) is an autosomal recessive disorder characterized by hearing impairment and delayed onset retinitis pigmentosa (RP) that affects 1 in 20,000 individuals worldwide (Keats and Corey 1999). Fifteen genes are associated with three clinical subtypes of Usher. Six genes are known to cause type 1 Usher (USH1) (Mathur and Yang 2015), the most severe form with profound sensorineural hearing impairment and vestibular are flexia at birth, and adolescent-onset RP. One form of Usher syndrome results from mutations in *USH1C*, which encodes Harmonin, a scaffolding protein that localizes to auditory hair cells and is essential for normal hair bundle morphogenesis and mechanotransduction (Verpy et al. 2000; Boeda et al. 2002; Lefevre et al. 2008; Grillet et al. 2009). Some mutations in the *USH1C* gene cause non-syndromic hearing impairment and others cause USH1 (Bitner-Glindzicz et al. 2000; Verpy et al. 2000). A mutation at position c.216G>A in exon 3 of the *USH1C* gene creates an aberrant splice site the use of which results in a truncated messenger RNA (mRNA) and Harmonin protein (Lentz et al. 2005) and is responsible for type 1C Usher (USH1C) in the Acadian population of Louisiana, USA and Canada. Similar to USH1C human patients, *Ush1c* c.216G>A knock-in mice (216AA) exhibit auditory, vestibular, and visual deficits. These Usher mice have little or no auditory evoked brainstem responses, exhibit circling behavior, and show abnormal electroretinograms (ERG) (Lentz et al. 2007). In the cochlea, peripheral defects in hair cell bundle morphology, as well as missing inner and outer hair cells, are present in 216AA mutant mice (Lentz et al. 2010; Lentz et al. 2013).

We have previously reported on a splice switching antisense oligonucleotide (ASO) (termed ASO-29) that base pairs to the 216A region and thereby blocks the recognition of the aberrant splice site. Remarkably, a single systemic dose of this ASO targeting the 216A mutation, administered a few days after birth, improves these peripheral defects and rescues hearing and circling behavior in 216AA mice (Lentz et al. 2013). A significant decrease in auditory-evoked brainstem response (ABR) thresholds was measured for several months after ASO treatment, indicative of the rescue of IHC function and hearing. Here, we investigate whether rescue of OHC function contributes to the hearing rescue by ASOs in Usher mice.

OHC function is commonly assessed by the measurement of otoacoustic emissions (OAEs) (Gold 1948; Kemp 1978, 1979), which are sounds produced by the inner ear in response to acoustic stimuli. The emissions are the result of the active process of OHCs to amplify the passive motion of the basilar membrane created by sound waves (Horner et al. 1985; Schrott et al. 1991; Dallos et al. 2008). When stimulated, OHCs elongate and shorten via the membrane protein prestin, amplifying basilar membrane displacement (Zheng et al. 2000; Liberman et al. 2002) and sharpening frequency discrimination. Conversely, the loss of OHCs results in a 50-dB threshold rise in audiograms with a loss of frequency selectivity in gerbils, chinchillas, macaque, and patas monkeys (Stebbins et al. 1969; Clark et al. 1974; Ryan and Dallos 1975; Moody et al. 1978; Stebbins et al. 1979; Smith et al. 1987).

Distortion product otoacoustic emissions (DPOAEs) are generated by the non-linear interaction of two tones at specific regions along the basilar membrane and reflect the function of the active process of OHCs. Here, we show that DPOAEs are absent in Usher mice but can be recovered by treatment with ASOs at post-natal day 1 (P1), an effect that is accompanied by a significant reduction in ABR thresholds, indicating a rescue of hearing. Treatment at P5 does not result in measureable DPOAEs, though significant reductions in ABR thresholds are detected, albeit not as reduced as with an earlier treatment time. Multiple administrations of ASOs beginning at P1 lead to more robust recovery of DPOAE and ABR responses. These results indicate that ASO-29 treatment can rescue IHC and OHC function and recovery is dependent on the timing of treatment, suggesting a developmental window during which expression of *Ush1c* and harmonin protein production is essential.

## **METHODS**

### Mice

*Ush1c* c.216G>A knock-in (216AA) and littermate mice were bred and treated at LSUHSC. All procedures met the NIH guidelines for care and use of laboratory animals and were approved by the Animal Care and Use Committee at LSUHSC. Mice were genotyped as described previously (Lentz et al. 2010).

### ASOs

Antisense oligonucleotides (2′-*O*-methoxyethyl-modified, Ionis Pharmaceuticals, Inc.) targeting the *Ush1c* c.216G>A mutation (ASO-29) (5′-AGCTGATCATATTCTACC-3′) and a non-specific control (ASO-C) (5′-TTAGTTTAATCACGCTCG-3′) were generated as previously described by (Lentz et al. 2013).

### Intraperitoneal Injection

*Ush1c* 216AA mutant and control 216GA mice were treated systemically by intraperitoneal injection with 300 mg/kg body weight of ASOs. For a single treatment regimen, mice were given one dose in the first week of life on post-natal day 1, 5, or 7. For a multiple treatment regimen, mice were given two doses in the first week of life on post-natal days 1 and 3 (P1, 3) or four doses on post-natal days 1, 3, 5, and 7 (P1, 3, 5, 7).

### Distortion Product Otoacoustic Emission Analysis

Distortion product otoacoustic emissions were measured to evaluate outer hair cell function in 216AA mice and controls at 1 month (P33–36), 3 months, and 6 months of age. Mice were anesthetized (intraperitoneal ketamine/xylazine, 100/6 mg/kg body weight, respectively), and body temperature was maintained near 38 °C with a heat pad. Primary tones f1 and f2 were selected to stimulate the low and mid-frequency regions of mouse auditory sensitivity. Primary tones (6363, 6672, 10,008, 13,342, 16,000, and 24,500 Hz) were presented at equal amplitude, with the ratio of f2 and f1 (f2/f1) equal to 1.199 (Harris et al. 1989; Martin et al. 2006). Stimulus amplitude was calibrated (± 1.5 dB) at the position of tympanum using continuous tones with a Bruel and Kjaer 2610 measuring amplifier (fast, linear weighting), 4135 microphone (grid on), and a 4230 pistonphone calibrator. Equal amplitudes of the two tone stimuli (f1 and f2) were checked with a dynamic signal analyzer (Hewlett Packard, 35106A); overall amplitude was controlled with TDT PA2 programmable attenuators and allowed to mix in the ear canal. The range of amplitudes tested was 35 to 75 dB sound pressure level (SPL) (re. 20 μPa). Tones were generated (195 kHz srate; 168 ms pulses with 0.5 ms linear ramps) using custom software and two EC1 (TDT) electrostatic speakers driven by ED1 electrostatic speaker driver. The stimuli were delivered to the left ear canal with an acoustic probe assembly (Entymotic Research, ER-10B+), tightly coupled to the ear. The ER-10B+ Low Noise Microphone System, also in the left ear canal, was used to detect the emission signal. The emission was digitized in 210 ms duration sweeps (7 μs; sampling period; 50 repetitions) and amplified (20 dB) using a TDT and System I array processor. Custom built MATLAB R2013a programs were used to compute the power of the emission signal using fast Fourier Transform (sampling frequency of 0.5 Hz) at respective 2f1-f2 frequencies. The noise floor was measured as the average power 100 Hz above and below the 2f1-f2 frequency. Thus, the DPOAE signal to noise ratio (SNR) is the amplitude at the 2f1-f2 frequency/noise floor. For plotting DPOAE thresholds, iso-response curves show the minimum f1 and f2 amplitude necessary to elicit a DPOAE (2f1-f2) with amplitude of 3 dB relative to the noise floor. Thus, elevated DPOAE thresholds reflect the case in which the noise floor has remained the same, but more stimulus power is required to produce the distortion product, reflecting reduced function of the active process. All experiments were conducted in a sound proof chamber. The signal-to-noise ratios were obtained for each of the pairs of tones tested. Data are reported as standard error of the mean.

### Auditory-Evoked Brain Stem Response Analysis

Hearing function was assessed as described by Lentz et al. (2013). Briefly, auditory-evoked brainstem responses (ABR) were used to evaluate hearing thresholds in *Ush1c* 216AA mutant and control mice at 1 month (P33–36), 3 months, and 6 months of age. Mice were anesthetized (intraperitoneal ketamine/xylazine, 100/6 mg/kg body weight, respectively), and body temperature was maintained near 38 °C with a heat pad. All acoustic stimuli were 5 ms pulses with 0.5 ms linear ramps. Tonal stimuli consisted of 8, 16, and 32 kHz to stimulate the low-, mid-, and high-frequency regions of basilar membrane. A broadband noise (BBN) was used to stimulate the whole cochlea. After amplification (Cambridge Audio Azur 540a Integrated amplifier), the stimuli were broadcast through a Motorola piezoelectric speaker (model no. 15D87141E02) fitted with a plastic funnel and 2 mm diameter tubing over the speaker front, producing an acoustic wave guide positioned in the external meatus approximately 0.5 cm from the tympanum. Using continuous tones, stimulus amplitude was calibrated at the end of the tubing with a Bruel and Kjaer 2610 measuring amplifier (fast, linear weighting), 4135 microphone (grid on), and 4230 pistonphone calibrator. All stimulus amplitudes were in dB SPL (re. 20 μPa). Stimuli were generated (195 kHz srate) and responses digitized (10 kHz srate) using TDT System III hardware and software (BioSig). ABRs were recorded with a 27 gauge subdermal steel electrode (Ambu Neuroline Subdermal) placed behind the left ear, with indifferent and ground electrodes (steel wire, 30 gauge) placed subcutaneously at the vertex and hind limbs, respectively. After amplification (60 dB, Grass P511 AC), filtering (0.3 Hz–1 kHz; TDT PF1), and averaging (*n* = 600–1024), thresholds (± 6 dB) were determined by visual inspection as the minimum stimulus amplitude that produced an ABR wave pattern similar to that produced for the highest intensity stimulus (90 dB). Data are reported as standard error of the mean.

We note that the ABR thresholds reported here are lower than those reported by Lentz et al. (2013). Although the source of this improvement is unclear, there was a difference in how mice were handled in this study compared to those in the previous one. In Lentz et al. (2013), mice were treated with the same dose (300 mg/kg) at P5 and subsequently shipped via air transport from Chicago to New Orleans where ABR analysis was performed. We hypothesize that eliminating this environmental stress could explain the more effective rescue measured here in locally housed and treated mice. Consistent with this hypothesis, we find that the normal (non-mutant) littermate controls that were shipped as part of the previous study also showed increased ABR thresholds compared to the data here.

### RNA Analysis

Inner ears were isolated and cochleae immediately frozen in liquid nitrogen or stored in Trizol reagent (Life Technologies). RNA was isolated from cochleae using Trizol reagent and analyzed by radioactive RT-PCR using primers musUSH1Cex2F (5′ CTCATTGAAAATGACGCAGAGAAGG 3′) and musUSH1Cex5R (5′ TCTCACTTTGATGGACACGGTCTT 3′) to amplify the correctly spliced (450 nt amplicon) or cryptic spliced (415 nt amplicon) *Ush1c* transcripts as previously described (Lentz et al. 2013). Briefly, 1 μg of RNA was reverse transcribed using GoScript Reverse Transcriptase (Promega, Fitchburg, WI) and 1 μg of cDNA was used in PCR reactions with GoTaq Green (Promega) supplemented with primers and α-^32^P-dCTP. Products were separated on a 6 % non-denaturing polyacrylamide gel and quantitated using a Typhoon 9400 phosphorimager (GE Healthcare). Data are reported as standard error of the mean. Statistical calculations were performed using Prism 6 version 6.0 h. The list of statistical comparisons and analyses can be found in Supplemental Table Comparison 5.

### Immunohistochemistry

Fluorescent labeling of microdissected preparations of the organ of Corti was used to study the localization of ASOs in mutant and heterozygote mice treated with ASOs or vehicle (saline) as described by Lentz et al. (2013). Briefly, a small opening was made in the apex of isolated cochleae and perfused with 2 % paraformaldehyde in 0.1 M phosphate buffer, pH 7.4 through the round and oval windows. The cochleae were post-fixed for 2 h at 4 °C with gentle rocking followed an overnight wash at 4 °C with PBS. The organs of Corti were then microdissected away from the cochlear bone, tectorial membrane removed with a fine forceps, and the stria vascularis trimmed. All incubations were performed with gentle rocking. Blocking and washing incubations were performed at room temperature and antibody incubations were performed at 4 °C. Tissues were immersed in Image IT (I36933, Thermo Fisher Scientific) for 30 min and then blocked for 2 h with 10 % normal donkey serum, 0.5 % bovine serum albumin, 0.03 % saponin, and 0.1 % Triton X-100 in PBS. Primary and secondary incubations were performed each for 2 h with 3 % normal donkey serum, 0.5 % bovine serum albumin, 0.03 % saponin, and 0.1 % Triton X-100 in PBS. To analyze ASO localization in cochlear hair cells, polyclonal rabbit anti-ASO antibodies (1:500; Ionis Pharmaceuticals) and mouse monoclonal anti-parvalbumin antibodies (1:250; P3088, Sigma-Aldrich) were used. Tissues were washed three times for 15 min each in 0.1 % Tween-20 in PBS after primary and secondary antibodies: donkey anti-rabbit Alexa555 (1:250; A31572, Thermo Fisher Scientific) and donkey anti-mouse Alexa488 (1:250; R37114, Thermo Fisher Scientific) incubations. Nuclei were counterstained with DAPI (1 μg/ml; D9542, Sigma-Aldrich). All labeled specimens were mounted and stored in Prolong Gold (Thermo Fisher Scientific). Imaging was performed with a Zeiss motorized system operated with LSM software (Zeiss) and equipped with 405-, 543-, and 633-nm diodes along with a multiline argon laser (457, 488, and 515 nm) and several objectives that include the Plan-Apochromat 40× (NA = 1.4 oil) and Plan-Apochromat 63× (NA = 1.4 oil). Planes were captured at a resolution of 2048 × 2048 pixels and speeds of 1–2 μs per pixel. Z-stack images were reconstructed and analyzed using ImageJ, Fuji, and Photoshop software.

### Statistical Analysis

ABR threshold, DPOAE thresholds, and DPOAE SNRs were analyzed in separate one-way analysis of variance models (ANOVA). Threshold or SNR was the outcome variable and gene, dose, stimulus intensity, and treatment were combined into a one-way set of combinations of predictive factors at each time point to deal with imbalances in the number of observations. ANOVA analysis was followed with a Tukey-Kramer adjustment for multiple comparisons which has good performance with unequal numbers of replicates (Kramer 1956). The reported *P* values are from alpha level adjusted *t* tests of differences between least-square means (Milliken and Johnson 1984). Differences were considered statistically significant when *P* ≤ 0.05. Statistical calculations were performed using Statistical Analysis System 9.4 software (SAS Institute Inc.). The list of statistical comparisons and analyses associated with ABRs and DPOAEs can be found in the Supplemental Table.

## **RESULTS**

### *Ush1c*^*216AA*^ Mice Lack Outer Hair Cell Function

**Fig. 1:**
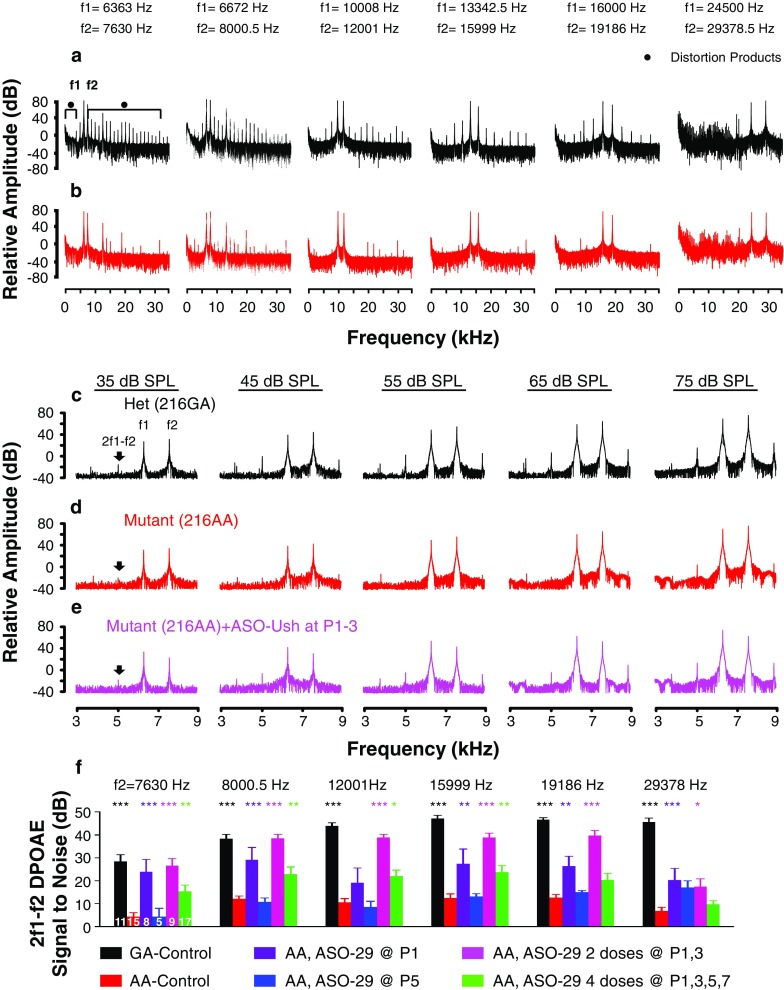
Distortion product optoacoustic emission analysis in ASO-treated *Ush1c* mice. Representative power spectra for all tone pairs at the highest stimulus intensity of 75 dB SPL from a **a** 216GA control littermate (black) and **b** *Ush1c* 216AA mouse (red). Power spectrum of the tone pairs at primary frequency tones of 6363 and 7630 Hz for *Ush1c* 216AA mice treated with 300 mg/kg of ASO-29 at P1–3 (**e**, pink line) and untreated 216AA (**d**, red line) and 216GA control (**c**, black line) mice at 1 month of age. The primary tones f1 and f2 and 2f1-f2 distortion product (black arrow) peaks are indicated. Response at 2f1-f2 increases with stimulus intensity for 216GA mice and 216AA mice treated with ASOs at P1–3 but not for untreated 216AA mice. **f** Average 2f1-f2 distortion product at 75 dB SPL for the tone pairs in 216AA mice treated with 300 mg/kg of ASO-29 with one dose at P1 (purple) or P5 (blue), two doses at P1 and 3 (P1, 3; pink), or four doses at P1, 3, 5, and 7 (P1, 3, 5, 7; green); 216AA untreated (red) and control littermates (black). The number of mice assayed is indicated in bars. Error bars represent SEM. For each frequency, asterisks indicate significant difference (*P ≤ 0.05, **P ≤ 0.01, ***P ≤ 0.001, ANOVA with Tukey-Kramer post-test) from control mutant thresholds (red). *P* values, specific test, test value, and degrees of freedom for all comparisons in this dataset are shown in Supplemental Table Comparison 1. dB, decibel; SPL, sound pressure level, kHz, kilohertz; Het, heterozygote; f, frequency

To test the function of the outer hair cells in the Ush1c^216AA^ mice (216AA), DPOAEs were measured at 1 month of age and compared to heterozygous Ush1c^216GA^ (216GA) mice. The power spectrum of the recorded otoacoustic emissions in response to tone pairs (f1 and f2) demonstrated that 216GA mice produce distortion products at 2f1-f2 and other harmonics (Fig. 1a, c). 216GA mice showed a strong signal at 2f1-f2 for the tone pairs in the low (6363, 7630 and 6672, 8000.5 Hz), mid (10,008, 12,001 and 13,342.5, 15,999 Hz), and high (16,000, 19,186 and 24,500, 29,378.5 Hz) frequencies. In contrast, 216AA mice had minimal or no distortion products (Fig. 1b). The 2f1-f2 distortion product at all of the tone pairs tested in 216AA mutant mice was significantly lower at a sound intensity of 75 dB SPL compared with 216GA mice (Fig. 1d, f). These results indicate that 216AA mice have a profound absence of OHC function.

### Age-Dependent Rescue of DPOAEs in ASO-Treated *Ush1c*^*216AA*^ Mice

To test whether ASO treatment can rescue OHC function in Ush1c^216AA^ mice, 216AA mice were treated with ASO-29 by intraperitoneal injection in the first week of life. Because OHC development continues post-natally, we injected the ASO at different times after birth to assess the requirement for treatment, and hence harmonin protein, during the development of OHCs. Dosing of mice with two or four injections of ASO every other day was also tested to maximize ASO delivery to cochlear hair cells. 216AA mice that had received a single ASO treatment at post-natal day 1 (P1) and multiple treatments at P1 and 3 (P1, 3) or at P1, 3, 5, and 7 (P1, 3, 5, 7) had significantly increased 2f1-f2 distortion products at 1 month of age at a sound intensity of 75 dB SPL for all tone pairs compared with untreated 216AA mice (Fig. 1e, f). A single ASO treatment given at P5, however, did not increase 2f1-f2 distortion products, suggesting that rescue of OHC function requires treatment before P5 either due to a requirement for harmonin expression before P5 or because less ASO reaches the hair cells when pups are treated with ASO at P5.

**Fig. 2:**
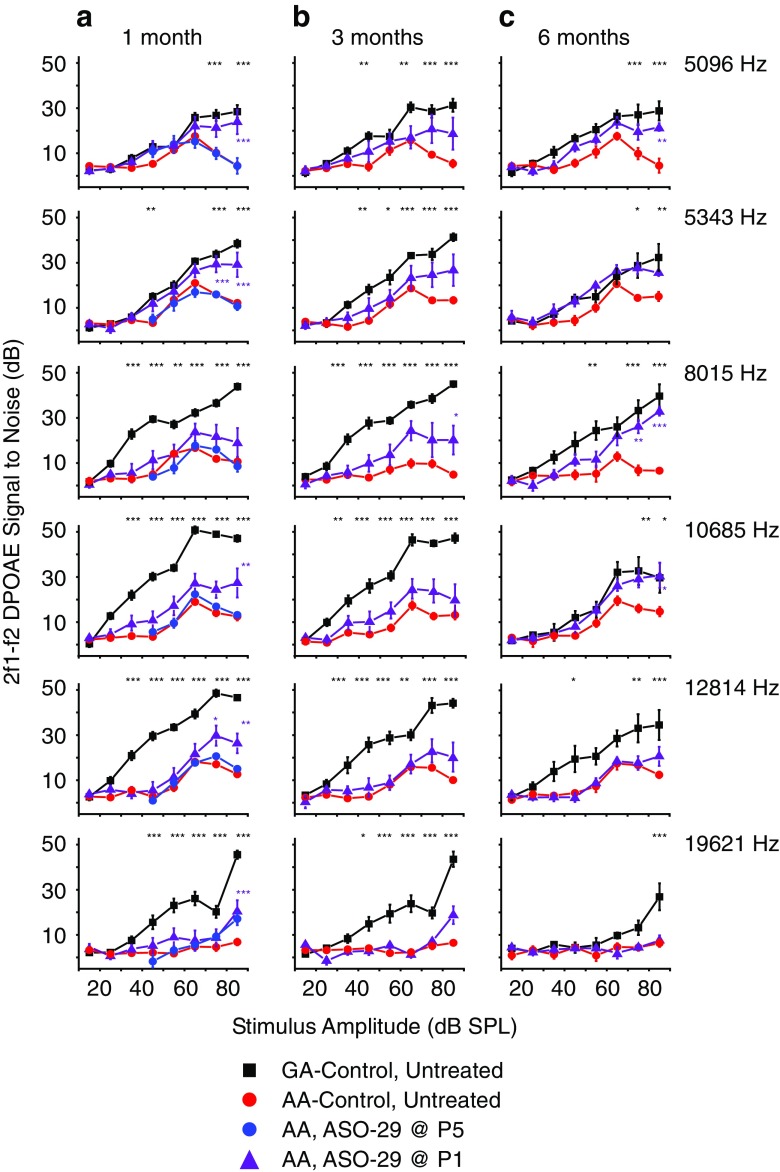
Analysis of DPOAE signal to noise ratios in *Ush1c* mice treated with a single dose of ASOs. Signal to noise ratio (SNR) plots at the primary tone pairs tested at 1 month (**a**), 3 months (**b**), and 6 months (**c**) of age for *Ush1c* 216AA mice treated with 300 mg/kg of ASO-29 one time at P1 (purple line, *n* = 8, 6, and 6 at 1, 3, and 6 months of age, respectively) or P5 (blue line, *n* = 5) and untreated 216AA (red line, *n* = 15, 12, and 7 at 1, 3, and 6 months of age, respectively) and untreated 216GA (black line, *n* = 11, 11, and 8 at 1, 3, and 6 months of age, respectively) control mice. Frequency tone pairs: f1 = 6363 Hz, f2 = 7630 Hz (2f1-f2 = 5096 Hz); f1 = 6672 Hz, f2 = 8000.5 Hz (2f1-f2 = 5243 Hz); f1 = 10,008 Hz, f2 = 12,001 Hz (2f1-f2 DP at 8015 Hz); f1 = 13,342.5 Hz, f2 = 15,999 Hz (2f1-f2 DP at 10685 Hz); f1 = 16,000 Hz, f2 = 19,186 Hz (2f1-f2 DP at 12814 Hz); f1 = 24,500 Hz, f2 = 29,378.5 Hz (2f1-f2 DP at 19621). SNRs are increased at some frequencies at all ages tested with a single ASO treatment at P1, but not P5. Error bars represent SEM. For each frequency, asterisks indicate significant difference (*P ≤ 0.05, **P ≤ 0.01, ***P ≤ 0.001, ANOVA with Tukey-Kramer post-test) from control mutant thresholds (red). *P* values, specific test, test value, and degrees of freedom for all comparisons in this dataset are shown in Supplemental Table Comparison 1. f, frequency; DP, distortion product; DPOAE, distortion product otoacoustic emission; Hz, Hertz; dB, decibel; SPL, sound pressure level; Het, heterozygote

**Fig. 3:**
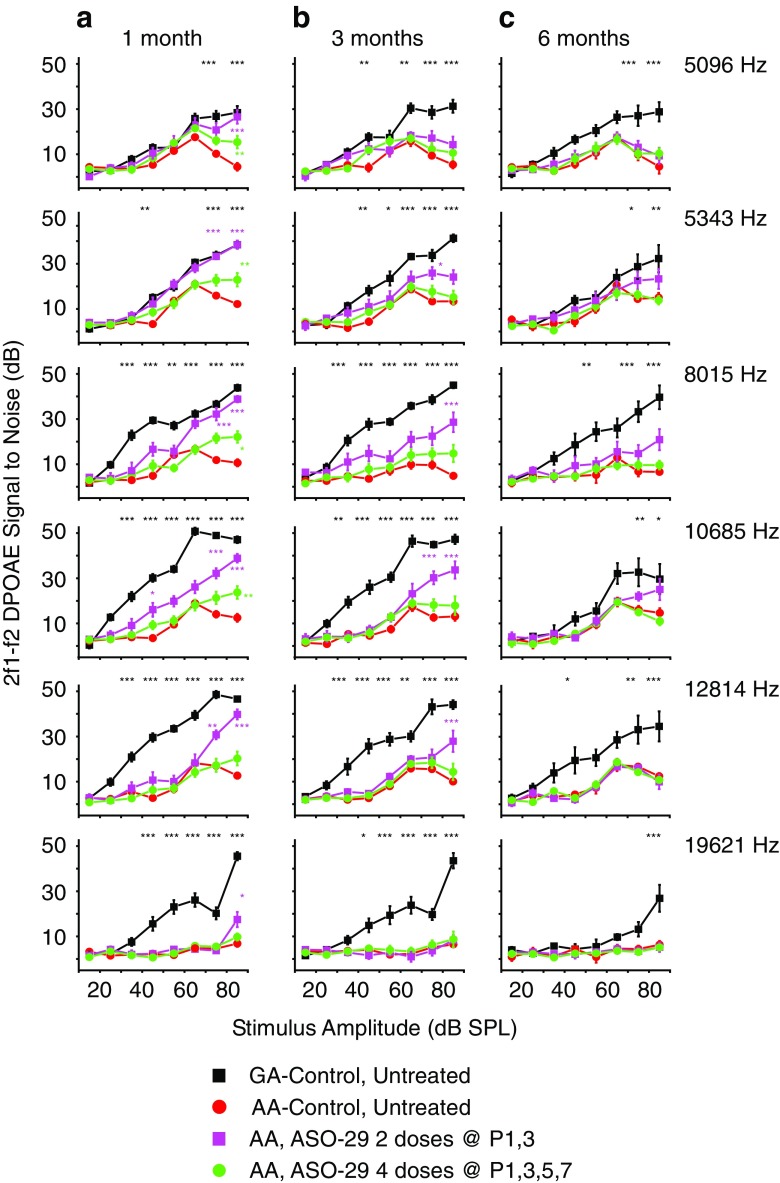
Analysis of DPOAE signal to noise ratios in *Ush1c* mice treated with multiple doses of ASOs. Signal to noise ratio (SNR) plots at the primary tone pairs tested at 1 month (**a**), 3 months (**b**), and 6 months (**c**) of age for *Ush1c* 216AA mice treated with 300 mg/kg of ASO-29 twice at P1 and 3 (P1, 3, pink line, *n* = 9, 8, and 7 at 1, 3, and 6 months of age, respectively) or four times at P1, 3, 5, and 7 (P1, 3, 5, 7, green line, *n* = 17, 13, and 12 at 1, 3, and 6 months of age, respectively) and untreated 216AA (red line, *n* = 15, 12, and 7 at 1, 3, and 6 months of age, respectively) and 216GA (black line, n = 11, 11 and 8 at 1, 3 and 6 months of age, respectively) control mice. Frequency tone pairs: f1 = 6363 Hz, f2 = 7630 Hz (2f1-f2 = 5096 Hz); f1 = 6672 Hz, f2 = 8000.5 Hz (2f1-f2 = 5243 Hz); f1 = 10,008 Hz, f2 = 12,001 Hz (2f1-f2 DP at 8015 Hz); f1 = 13,342.5 Hz, f2 = 15,999 Hz (2f1-f2 DP at 10685 Hz); f1 = 16,000 Hz, f2 = 19,186 Hz (2f1-f2 DP at 12814 Hz); f1 = 24,500 Hz, f2 = 29,378.5 Hz (2f1-f2 DP at 19621). SNRs are increased at some frequencies at 1 and 3 months of age with multiple ASO treatments in the first week of life. The number of treatments (doses) is indicated. Error bars represent SEM. For each frequency, asterisks indicate significant difference (*P ≤ 0.05, **P ≤ 0.01, ***P ≤ 0.001, ANOVA with Tukey-Kramer post-test) from control mutant thresholds (red)*. P* values, specific test, test value, and degrees of freedom for all comparisons in this dataset are shown in Supplemental Table Comparison 1 *.* f, frequency; DP, distortion product; DPOAE, distortion product otoacoustic emission; Hz, Hertz; dB, decibel; SPL, sound pressure level; Het, heterozygote

In order to test the stability of the effect of ASO-29 treatment on OHC function, DPOAEs were measured at 1, 3, and 6 months after treatment. The DPOAE signal to noise ratio (SNR) at 2f1-f2 for the 216GA control mice at 1, 3, and 6 months of age increased with increasing intensity levels (Figs. 2 and 3). By comparison, the SNRs for untreated 216AA mutant mice relative to those in 216GA littermates were low at all intensities and frequencies at all ages tested. However, a single ASO treatment at P1 (Fig. 2) and multiple ASO treatments at P1, 3 and P1, 3, 5, 7 (Fig. 3) significantly increased 2f1-f2 SNRs with increasing stimulus intensity levels. For these treatments, responses to stimulus intensities were significantly elevated at 1 month of age for all frequencies except 8015 Hz in mice treated at P1 (Fig. 2a) and 12,814 and 19,621 Hz in mice treated at P1,3,5,7 (Fig. 3a). There was no difference in SNRs at 1 month of age between single and multiple ASO treatments at P1 and P1, 3, except at 8015 Hz, in which P1, 3 treatments resulted in a significant increase in SNR (75 dB SPL; Supplemental Table Comparison 1). There was, however, a significant difference in SNRs at all frequencies except for the frequency pair at 19621 Hz between two (P1, 3) and four doses (P1, 3, 5, 7) of ASO with the largest increase in SNR observed in mice treated at P1, 3. At 1 month of age, a single ASO treatment in 216AA mice at P5 resulted in no change in DPOAE 2f1-f2 SNRs at all intensities at all frequencies relative to untreated 216AA mice (Figs. 1d and 2a).

At 3 months of age, the significant increase in SNR was sustained at 8015 Hz for 216AA mice treated with ASOs at P1 (Fig. 2b) and at multiple low-mid-frequency pairs for those treated at P1, 3 (Fig. 3b). Interestingly, a single dose of ASO-29 at P1 was the only treatment regimen that had a sustained significant increase in SNRs at low-mid frequencies at 6 months of age (Figs. 2c and 3c).

**Fig. 4:**
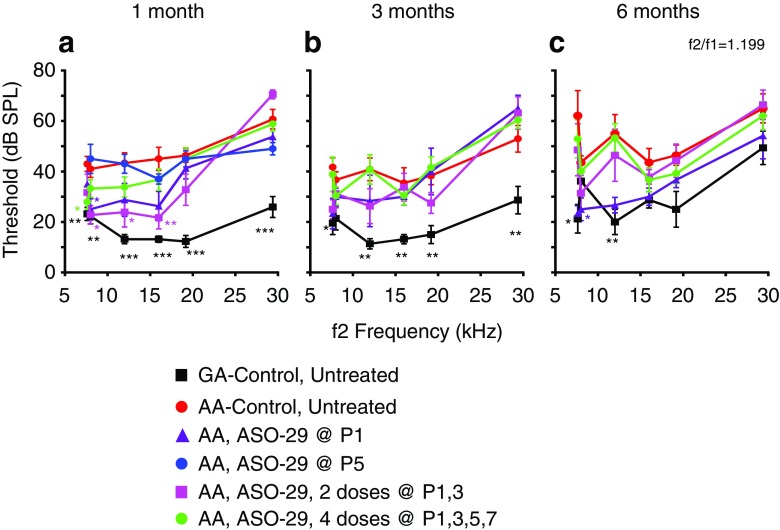
Distortion product otoacoustic emissions analysis in *Ush1c* mice treated with ASOs. Average DPOAE thresholds (dB SPL) at 1 month (**a**), 3 months (**b**), and 6 months (**c**) of age to tone pairs ranging from 6363 to 29,378.5 kHz from 216AA mice treated with 300 mg/kg of ASO-29 one time at P1 (purple line, *n* = 8, 6, and 6 at 1, 3, and 6 months of age, respectively) or P5 (blue line, *n* = 5) or multiple times at P1–3 (pink line, *n* = 9, 8, and 7 at 1, 3, and 6 months, respectively) or P1–7 (green line, *n* = 17, 13, 12 at 1, 3 and 6 months of age, respectively); 216AA mice treated with ASO-C or untreated (red line, n = 15, 12, 7 at 1, 3 and 6 months of age, respectively); and 216 untreated GA mice (black line, *n* = 11, 11, 8 at 1, 3, and 6 months of age, respectively). DPOAE thresholds are reduced with some ASO treatments in 216AA mice. Total number of treatments (doses) is indicated. Error bars indicate SEM. For each frequency, asterisks indicate significant difference (*P ≤ 0.05, **P ≤ 0.01, ***P ≤ 0.001, ANOVA with Tukey-Kramer post-test) from control mutant thresholds (red). *P* values, specific test, test value, and degrees of freedom for all comparisons in this dataset are shown in Supplemental Table Comparison 2. dB, decibels; SPL, sound pressure level; kHz, kilohertz; BBN, broad-band noise; GA, *Ush1c* c.216GA littermate controls, AA, *Ush1c* c.216AA

At 1, 3, and 6 months of age, DPOAE thresholds at all frequency pairs tested were significantly elevated in 216AA mutant mice compared with 216GA normal controls (Fig. 4). Treatment of 216AA mice with one dose at P1 or two doses (at P1 and 3) of ASO-29 resulted in significantly lower DPOAE thresholds at 1 month of age at low (f1/f2 = 6.672/8 kHz tone pair). Treatment at P1, 3 also resulted in a significantly lower DPOAE threshold at 1 month of age at mid frequencies (f1/f2 = 10/12 and 13.342/16 kHz tone pairs), whereas four doses (at P1, 3, 5, and 7) resulted in a significantly lower DPOAE threshold at 1 month of age at low frequency (f1/f2 = 6.363/7.63 kHz) compared to mutant controls (Fig. 4a). The reduction in DPOAE thresholds at all frequencies in ASO-treated 216AA mice, however, was not sustained at 3 and 6 months of age, with the exception of a significantly lower DPOAE threshold at low frequency (f1/f2 = 6.363/7.63 kHz) in 6-month-old 216AA mice treated once at P1 (Fig. 4b, c). This finding supports our results from DPOAE SNR measurements that show no significant effects at amplitudes below 70 dB across frequencies (Figs. 2 and 3). 216AA mutant mice treated at P5 had little or no distortion products and were similar to untreated 216AA mutant mice (Fig. 4b, c). Taken together, these data demonstrate that initiation of ASO treatment prior to P5 is required for rescue of the 2f1-f2 distortion product and multiple treatments may provide some additional early benefits, which are not sustained at later points of treatment.

### Age-Dependent Improvement in ABR Thresholds in ASO-treated *Ush1c*^*216AA*^ Mice

**Fig. 5:**
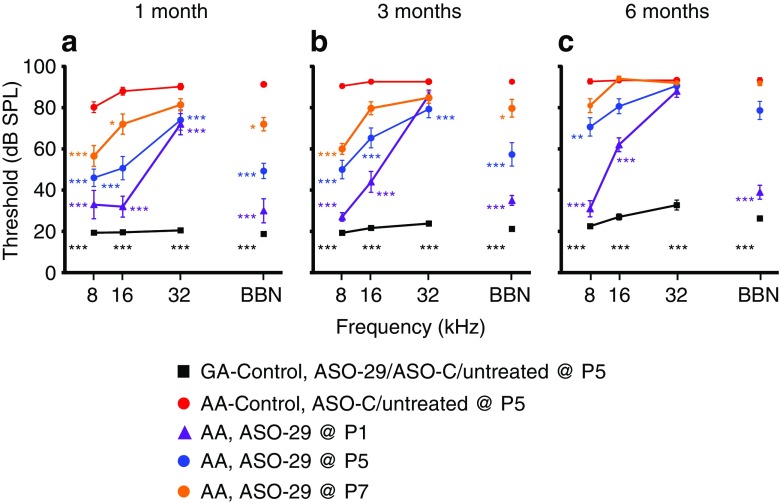
Auditory evoked brainstem analysis in Ush1c mice treated with a single dose of ASOs**.** Average ABR thresholds (dB SPL) at 1 month (**a**), 3 months (**b**), and 6 months (**c**) of age to pure tones ranging from 8 to 32 kHz or BBN from 216AA mice treated with 300 mg/kg of ASO-29 one time at P1 (purple line, *n* = 6, 6, and 6 at 1, 3, and 6 months of age, respectively), P5 (blue line, *n* = 9, 9, and 9 at 1, 3, and 6 months of age, respectively) or P7 (orange line, *n* = 7, 7 and 6 at 1, 3, and 6 months of age, respectively); 216AA mice treated with ASO-C or untreated (red line, *n* = 27, 23 and 11 at 1, 3 and 6 months of age); and 216GG/GA mice treated by IP injection with ASO-29, ASO-C, and untreated (black line, *n* = 31, 28, and 24 at 1, 3, and 6 months of age, respectively). ABR thresholds are reduced at some frequencies at all ages in 216AA mice treated with a single dose of ASOs in the first week of life. Error bars indicate SEM. For each frequency, asterisks indicate significant difference (*P ≤ 0.05, **P ≤ 0.01, ***P ≤ 0.001, ANOVA with Tukey-Kramer post-test) from control mutant thresholds (red). *P* values, specific test, test value, and degrees of freedom for all comparisons in this dataset are shown in Supplemental Table Comparisons 3 and 4. dB, decibels; SPL, sound pressure level; kHz, kilohertz; BBN, broad-band noise; GG/GA, *Ush1c* c.216GG/GA littermate controls, AA, *Ush1c* c.216AA

**Fig. 6:**
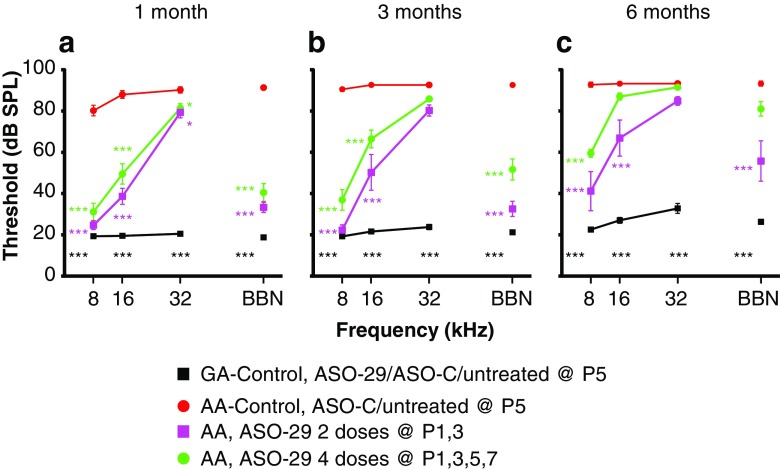
Auditory evoked brainstem analysis in *Ush1c* mice treated with multiple doses of ASOs. Average ABR thresholds (dB SPL) at 1 month (**a**), 3 months (**b**), and 6 months (**c**) of age to pure tones ranging from 8 to 32 kHz or BBN from 216AA mice treated with 300 mg/kg of ASO-29 twice at P1 and 3 (P1–3, pink line, *n* = 9, 7, and 7 at 1, 3 and 6 months of age) or four times at P1, 3, 5, and 7 (P1–7, green line, *n* = 16, 13, and 12 at 1, 3, and 6 months of age); 216AA mice treated with ASO-C or untreated (red line, *n* = 27, 23, and 11 at 1, 3, and 6 months of age, respectively); and 216GG/GA mice treated by IP injection with ASO-29, ASO-C, and untreated (black line, *n* = 31, 28, and 24 at 1, 3, and 6 months of age, respectively). ABR thresholds are reduced at some frequencies at all ages in 216AA mice treated with multiple doses of ASOs in the first week of life. Error bars indicate SEM. For each frequency, asterisks indicate significant difference (*P ≤ 0.05, **P ≤ 0.01, ***P ≤ 0.001, ANOVA with Tukey-Kramer post-test) from control mutant thresholds (red). *P* values for additional comparisons in this dataset shown in Supplemental Table Comparisons 3 and 4. Total number of treatments (doses) is indicated. dB, decibels; SPL, sound pressure level; kHz, kilohertz; BBN, broad-band noise; GG/GA, *Ush1c* c.216GG/GA littermate controls, AA, *Ush1c* c.216AA

To measure the effect of the different ASO-29 treatment regimens on hearing rescue, ABR analysis was performed at 1, 3, and 6 months of age on 216AA mutant mice treated with single or multiple doses of the ASO at different times in the first week of life, as described above. ABR thresholds of untreated or control-treated 216AA mice were significantly elevated at 1, 3, and 6 months of age for all frequencies tested (Lentz et al. 2010, 2013; Figs. 5 and 6). Single and multiple ASO treatments in the first week of life in 216AA mice rescued ABR thresholds, with earlier intervention and multiple treatments resulting in the lowest thresholds (Figs. 5 and 6).

At 1 month of age, ABR thresholds were significantly reduced in 216AA mice for all ASO treatments at low (8 kHz) and mid (16 kHz) frequency pure tones and broadband noise (BBN) stimuli when compared with control-treated 216AA mice (Figs. 5a and 6a). Furthermore, the ABR thresholds of P1- and P1, 3-treated 216AA mice were not significantly different at low and mid frequencies compared with their normal hearing 216GA heterozygous littermates (Supplemental Table Comparison 3). For high-frequency stimuli (32 kHz), ABR thresholds in ASO-treated 216AA mice were significantly reduced compared with 216AA controls for all ASO treatments except in mice treated at P7 (Fig. 5a). When a single dose of ASOs was given as late as P7, low-mid-frequency hearing, but not high-frequency hearing, was rescued. At 8 kHz, ABR thresholds after a single ASO treatment at P1 were significantly different from treatment at P7, but not P5. At 16 kHz, ABR thresholds after a single ASO treatment at P1, 5, or 7 were significantly different from each other, with P1 having the lowest thresholds among all three treatment ages. There was no significant difference in ABR thresholds between the three treatment ages at 32 kHz. Multiple ASO treatments in 216AA mice at P1 and 3 had the lowest ABR thresholds at 8 kHz (24 dB SPL; Fig. 6a). However, there was no significant difference in thresholds between a single treatment at P1 (33 dB SPL; Fig. 5a) and two treatments at P1 and 3 (25 dB SPL; Fig. 6a) at 8 kHz. Among the multiple treatment regimens (two doses versus four doses), there were no significant differences in ABR thresholds at 1 month of age for all the stimuli tested.

At 3 months of age, ABR thresholds in response to low-frequency stimulation (8 kHz) remained significantly lower in 216AA mice for all ASO treatments compared with mutant controls (Figs. 5b and 6b). For mid-frequency (16 kHz) and BBN stimuli, ABR thresholds remained significantly lower compared with 216AA control-treated mice for all ASO treatments except for P7. Additionally, ABR thresholds of P1- and P1, 3-treated 216AA mice were not significantly different for 8 kHz and BBN stimulus (P1, 3) compared with heterozygous littermates. For the high-frequency (32 kHz) stimulus, a single treatment at P5 and 2 treatments at P1, 3 had ABR thresholds that remained significantly reduced compared with 216AA controls.

There was no significant difference in ABR thresholds between 1- and 3-month-old ASO-treated 216AA mice for all frequencies and all treatments except at 16 kHz in 216AA mice treated with four doses (P1, 3, 5, 7) (Figs. 5a, b and 6a, b). Remarkably, ABR thresholds in response to low-frequency stimulation (8 kHz 33, 27, and 31 dB SPL at 1, 3, and 6 months of age, respectively; Fig. 5) and BBN stimulus (30, 35, and 39 dB SPL at 1, 3, and 6 months of age, respectively; Fig. 5) in 216AA mice treated one time at P1 were stable until 6 months of age, remaining significantly lower compared with 216AA controls (Fig. 5c). For all other ASO treatments and frequencies tested, ABR thresholds were elevated at 6 months of age (Figs. 5c and 6c). This elevation notwithstanding, ABR thresholds in mice that underwent all treatment regimens remained significantly lower compared to control 216AA mice except those treated at P7. ABR thresholds in response to low-frequency stimulation (8 kHz) were significantly lower compared with 216AA controls for a single ASO treatment at P1 or P5 and multiple treatments at P1, 3 or P1, 3, 5, 7 (Figs. 5c and 6c). For mid-frequency (16 kHz) and BBN stimuli, ABR thresholds in mice treated at P1 and P1, 3 remained significantly lower compared with 216AA control-treated mice. For the high-frequency (32 kHz) stimulus, ABR thresholds were not significantly different in ASO-treated 216AA mice compared with 216AA controls. For single treatments, 6-month ABR thresholds were significantly lower at low-mid (8 and 16 kHz) frequency and BBN stimulus in 216AA mice treated at P1 compared to P5 and P7 (Supplemental Table Comparison 3). For multiple treatments, ABR thresholds were significantly lower at low-mid (8 and 16 kHz) frequency and BBN stimulus in 216AA mice treated with 2 doses at P1, 3 compared to 4 doses at P1, 3, 5, and 7. ABR thresholds were not significantly different for low-frequency stimulation (8 kHz) and BBN stimulus in P1-treated 216AA mice compared with 216GA heterozygous littermates. Although there was variance associated with stimulus frequency and duration of effect, these data demonstrate that all treatment regimens resulted in ABR threshold rescue.

### Correction of *Ush1c* c.216A Splicing in the Cochleae of ASO-Treated Mice

**Fig. 7:**
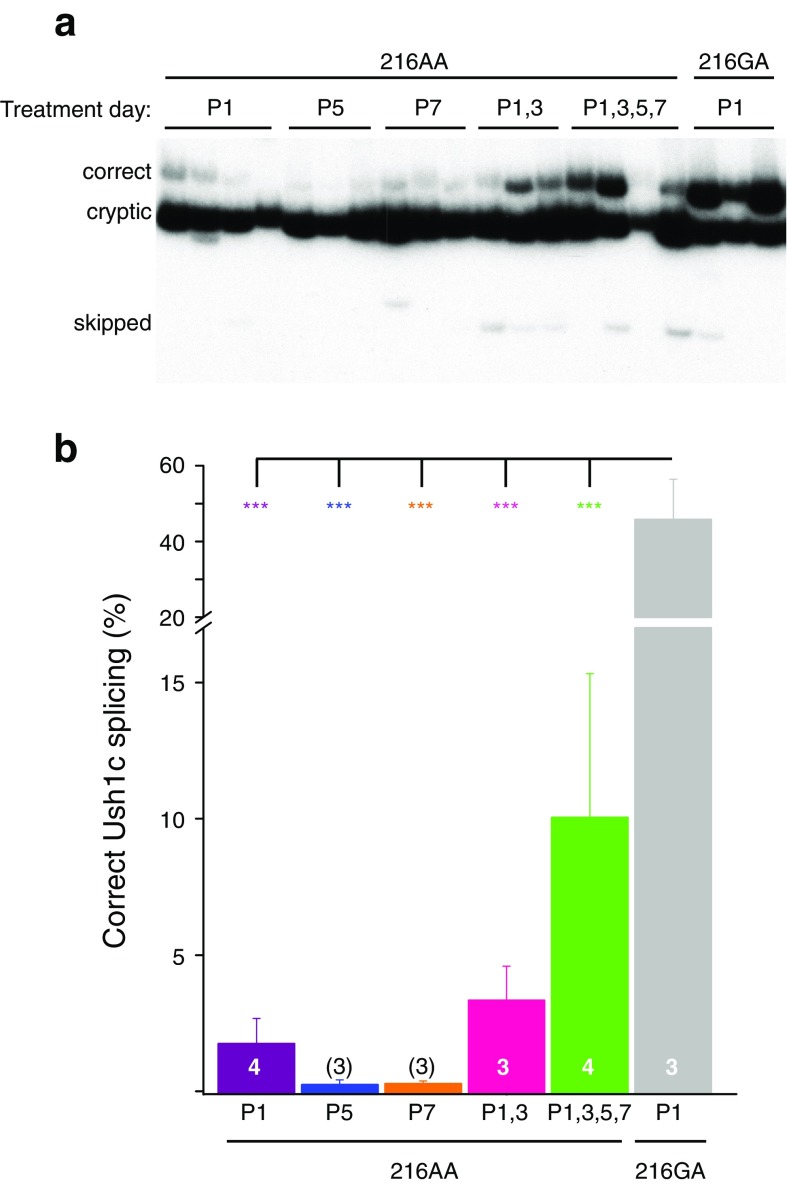
ASO-mediated correction of *Ush1c* c.216A pre-mRNA splicing 6 months post-injection. **a** RT-PCR analysis of cochlear RNA isolated from 6-month-old mice treated with 300 mg/kg of ASO-29 at indicated post-natal day(s). Spliced products are labeled. **b** Quantitation of PCR products shown in **a**. Asterisks indicate significant difference (***P ≤ 0.001, one-way ANOVA, Tukey’s multiple comparison test)

To determine the effect of the number and timing of ASO-29 treatments on *Ush1c* c.216A splicing, *Ush1c* mRNA was isolated from cochlear tissue that was harvested from 6-month-old 216AA mice treated with ASOs once at P1, P5, or P7 or with two doses at P1 and 3 (P1, 3) or four doses at P1, 3, 5, and 7 (P1, 3, 5, 7). RT-PCR analysis of cochlear mRNA revealed that a small percent of the total *Ush1c* c.216A mRNA was correctly spliced (Fig. 7). The amount of correctly spliced mRNA was highest in the cochleae from mice treated with multiple doses of the ASO. The relative abundance of correctly spliced mRNA was not significantly different among the treatment regimens, suggesting that the delivery of the ASO to the cochlea is similar at the different treatment ages and results in a similar correction of splicing (Fig. 7). Overall, our results suggest that treatment prior to P5 is critical for the rescue of OHC function likely due to a temporal requirement for harmonin rather than a result of temporal differences in ASO delivery.

### Localization of ASO-29 in *Ush1c* Cochlear Hair Cells

**Fig. 8:**
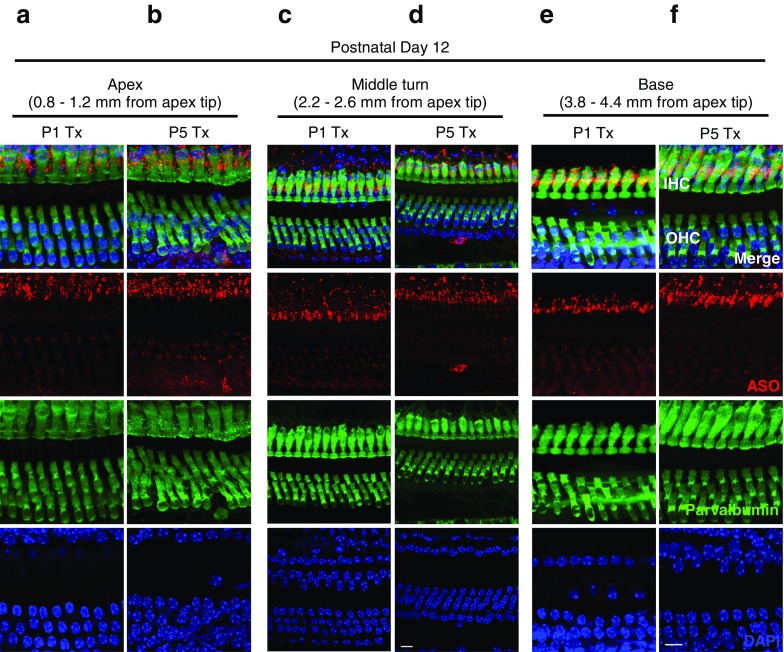
Localization of ASOs in auditory hair cells at P12 after systemic treatment in *Ush1c* mice**.** Immunofluorescent labeling of ASO-29 (red) in HCs (green) at the **a**, **b** apex, **c**, **d** middle turn, and **e**, **f** base at P12 after ASO treatment at P1 or P5. Distance from the apex tip is indicated. Scale bars indicate 10 μm. IHC, inner hair cell; OHC, outer hair cell; ASO, antisense oligonucleotide

**Fig. 9:**
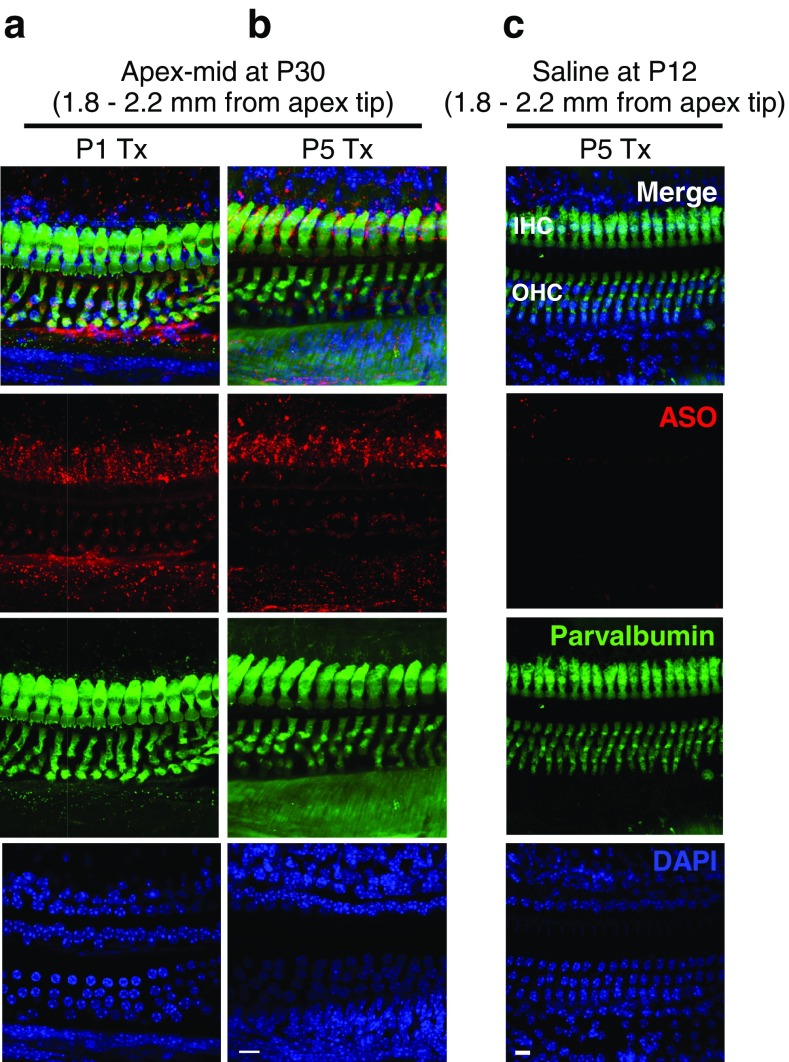
Localization of ASOs in auditory hair cells at P30 after systemic treatment in *Ush1c* mice**.** Immunofluorescent labeling of ASO-29 (red) in HCs (green) at the **a**, **b** apex-mid turn at P30 after ASO treatment at P1 (**a**) or P5 (**b**). **c** Immunofluorescent image at the apex-middle turn of P5 vehicle-treated (saline), control littermate 1 week after P5 ASO treatment (P12). Scale bars indicate 10 μm. IHC, inner hair cell; OHC, outer hair cell; ASO, antisense oligonucleotide

To determine whether intraperitoneal ASO treatment results in localization of ASO to both HC populations, we performed immunofluorescence analysis of the organs of Corti of treated *Ush1c* mice using an antibody that specifically recognizes the ASO. 216AA mice were treated with ASO-29 or saline at P1 or P5 and tissues harvested at P12 and P30. At P12, ASO (red puncta) was detected in 216AA mutant IHCs and OHCs at the apex, mid, and basal regions of the cochlea (Fig. 8). ASO-29 was still abundant in both IHCs and OHCs of treated mice at 1 month of age (Fig. 9a, b). ASO-29 was also observed surrounding the HCs in a pattern suggesting localization within support cells. Saline-treated control littermates displayed little or no red puncta, demonstrating the specificity of the antibody for the ASO (Fig. 9c). These results demonstrate the presence of ASO-29 in the hair cells and indicate that there is similar localization of ASO to HCs in P12 and P30 mice that were treated at P1 or P5 with ASO-29.

## **DISCUSSION**

In this study, we have identified a critical time at which treatment must occur in order to rescue IHC and OHC function in a mouse model of Usher syndrome using ASOs that correct *Ush1c* c.216A gene expression. Our previous work with *Ush1c* c.216G>A (216AA) mice demonstrated that although mutant mice exhibit little or no hearing and abnormal balance during locomotion (Lentz et al. 2007), ASO-29 treatment can rescue auditory function and eliminate circling behavior (Lentz et al. 2013). In particular, when compared with control- and untreated-mutant ABRs, measures of auditory sensitivity demonstrated a threshold reduction of ~ 44 dB in ASO-treated mutant mice. However, it was yet to be determined if this increase in sensitivity was due to improved IHC and OHC function or limited to IHCs alone. Indeed, the rescued ABR thresholds remained above those of wild-type controls, raising the possibility that therapeutic effects of the ASO may be limited to IHC mechanisms, unaided by the active process. Thus, here we utilize a functional measure of the active process, DPOAE, to quantify the effect of ASO treatment on OHC function.

Our results show that 216GA heterozygous mice, which have ABR thresholds similar to wild-type mice, also exhibit typical DPOAEs with measurable distortion products in response to two tones of a given frequency and sound pressure level. In contrast, 1-month-old homozygous 216AA mutant mice exhibit profoundly attenuated ABRs and have little or no DPOAEs. A single systemic ASO treatment at P5 rescued low- and mid-frequency ABRs but did not rescue DPOAEs suggesting that ASO-29 can rescue IHC, but not OHC function. However, treatment with ASO-29 earlier than P5, including a single dose at P1 or multiple doses beginning at P1, significantly improved both ABRs and DPOAEs, suggesting that OHC function can be rescued by ASO-29. Although ABR and DPOAE thresholds were lower with two doses at P1 and 3 compared to a single dose at P1, there was no significant difference between them, suggesting little added benefit from an additional dose at P3. Interestingly, four doses of ASO-29 at P1, 3, 5, and 7 were not as effective at rescuing DPOAEs or ABRs compared to a single treatment at P1 or two doses at P1 and P3 (Figs. 1, 3, and 4) despite a trend towards a higher level of splicing correction (Fig. 7). We hypothesize that the most important dosing is at P1 and that harmonin expression between P1 and P3 is critical for the HC functions that we measured. The explanation for the lack of increased efficacy with this treatment regimen is not clear. It is possible that repeated IP dosing early in life has specific or non-specific toxic effects in the animal that affect IHC and OHC function. Overall, our results demonstrate the importance of early treatment for rescue of OHC function, which is consistent with a recent report that DPOAE thresholds could be rescued in 216AA mice following delivery of wild-type *Ush1c* to the inner ear in P1 pups using an adeno-associated viral vector (Pan et al. 2017).

It is possible that the lack of rescue of the OHC active process when ASO is delivered at P5 is a result of differences in the extent to which ASOs reach IHCs and OHCs. However, immunofluorescent analysis of ASO distribution in the cochlea ~ 1 week after treatment indicates that ASO is present in both IHCs and OHCs, though apparently more abundant in IHCs (Fig. 8). By 1 month of age, approximately 3.5 weeks after treatment, when hearing assessment was performed, ASO was present in both IHCs and OHCs in areas that correspond to low- and mid-frequency hearing (Fig. 9). The morphological data notwithstanding, the quantity of ASO reaching the OHCs and the timing of ASO-29 treatment may be expected to vary between hair cell types as their precursor cells and developmental times vary (Ruben 1967; Rubel 1978; Pirvola et al. 1991; Lim and Rueda 1992; Muller and Littlewood-Evans 2001; Liberman et al. 2002; Okoruwa et al. 2008; Ahmed et al. 2012; Burns et al. 2012). Furthermore, exposure within cell types may vary with time. Indeed, development of OHC transduction currents proceed basal to apical (P0-P2), making the timing of the ASO therapy at P5 more closely synchronized to OHC development in low-frequency regions than high-frequency basal turns (Waguespack et al. 2007). In either case, treatment with ASOs at P5 is likely subsequent to much of the OHC transduction development, suggesting that the failure of OHC rescue could be overcome with earlier ASO delivery.

In addition to their different morphologies, functions and development, vestibular hair cells, IHCs, and OHCs vary in their response to different genetic deletions (Friedman et al. 2007; Dror and Avraham 2010; Fritzsch et al. 2013). The results here, showing a failure of ASO-29 to rescue OHC function at P5 (but success in IHC and vestibular function as previously reported Lentz et al. 2013 and Vijayakumar et al. 2017), suggest that the function of harmonin, the amount expressed, and/or the cells sensitivity to mutant protein levels may be relevant to these distinguishing characteristics. Thus, not only do the data here create a framework of understanding both the disease and therapeutic mechanisms, but they also contribute to our knowledge of the differences in the underlying proteomic mechanisms differentiating these cell types.

With respect to heritable deafness mutations, evidence in humans also suggests differences in IHC and OHC mechanisms. Humans with Usher syndrome and other forms of hearing loss present with hearing deficits consistent with IHC and OHC functional loss (Shinkawa and Nadol 1986; Cohn et al. 1999; Wagenaar et al. 2000). Interestingly, while pure tone ABR thresholds are similar, differences in OAEs between carriers and non-carriers of mutations that cause several different types of hearing impairment are observed, including carriers of Usher syndrome type 1C, some carriers of the 35delG mutation in Connexin 26 (Cx26), Ashkenazi Jewish carriers of the 167delT mutation in Cx26, DFNA11, and autosomal dominant non-syndromic hearing impairment caused by a mutation in the myosin VIIA gene (Morell and Friedman 1999; Engel-Yeger et al. 2002; Hood et al. 2002; Tamagawa et al. 2002; Engel-Yeger et al. 2003; Franze et al. 2005). Thus, our data are consistent with the phenotypes of these diseases, as our data also suggest different underlying mechanisms of the mutation in IHCs and OHCs.

The current study establishes the critical time in the development of hearing at which treatment must occur in order to correct both IHC and OHC dysfunction in Usher syndrome type 1C. We have established that ASOs are an effective drug platform to achieve hearing rescue for a human form of congenital deafness in mice. ASOs have emerged as a powerful therapeutic for a number of different diseases and conditions in humans, some of which have been approved for clinical use (Gryn and Hegele, 2015; Havens and Hastings, 2016; Finkel et al. 2016). From a therapeutic perspective for type 1 Usher syndrome, ASO-mediated treatment of hearing would likely require a very early intervention point in humans, where hair cell development occurs early in gestation in utero. We have recently reported on the ability of ASOs to access the cochleae of mice exposed to the molecule via injection into the amniotic cavity (Depreux et al. 2016). This result suggests that early treatment with ASOs in utero is feasible in mice and could be utilized as a means to deliver a potential ASO drug during the critical period for hearing development.

## Electronic Supplementary Material

Supplemental Table 1 (XLSX 51 kb).
